# Diversity, preventive work and education—matters of health and well-being in firefighter discourse

**DOI:** 10.1080/17482631.2020.1817661

**Published:** 2020-09-16

**Authors:** Ann Jacobsson, Susann Backteman-Erlanson, Annika Egan Sjolander

**Affiliations:** a Department of Nursing, Umea University, Umea, Sweden; b Department of Culture and Media Studies, Umea University, Umea, Sweden

**Keywords:** Health, well-being, critical discourse analysis, firefighters, critical policy analysis, diversity, preventive work, education

## Abstract

**Purpose:** The aim of this study is to analyse how recurrent health hindrance themes in the firefighter discourse, identified by firefighters themselves, relate to a set of policies about diversity, preventive work and education of firefighters. The intention is further to discuss the implications of these policy initiatives and the resistance against them in terms of firefighters’ health and well-being at work.

**Method:** Firefighters from three different rescue stations in Sweden, participated in either a focus group discussion or individual interviews. Different themes in firefighter discourse that were described as hindrances to the health and well-being of firefighters were identified. A strategic sample of policy documents that relate to the very same themes was also chosen for analysis and here we combined critical discourse analysis (CDA) with critical policy analysis.

**Results:** The health hindrance themes regarding diversity, preventive work and education that firefighters identified have in common that they relate to changes in work culture and the firefighter profession.

**Conclusion:** In conclusion, we argue that the most important challenge for the rescue service to tackle in the future, is how to transform firefighting to be more inclusive and yet maintaining the good health and well-being that exists among the vast majority of today’s firefighters.

## Introduction

This article focuses on firefighters’ health and well-being at work and in particular the part of firefighters’ work that seldom receives attention research-wise, namely the “ordinary” day to day activities and work culture at the rescue service station which in fact takes up most of the time in a firefighters working life. This is a little surprising, since emergency or operational work only takes up a small part of firefighters’ time as employees, even if these moments are often critical. Previous research about firefighters’ health and well-being in relation to their work concentrates mostly on the consequences of physically and psychologically stressful critical incidents; in other words situations when firefighters are considered as a high-risk group for mental ill-health (Benedek et al., 2007; Corneil et al., 1999). As far as we know, research about relationships between health and work among firefighters, when considering the organizational culture and the meaning-making processes of all the “ordinary”, day-to-day work at rescue stations, is scarce. In a previous study in this research project, we therefore explored how firefighters themselves reasoned about their health and well-being at work, and identified both recurrent promoting themes and hindrance themes (Jacobsson, Backteman-Erlanson, Brulin & Egan Sjolander, 2020, submitted manuscript). Based on the results from these focus group discussions (FGDs) and individual interviews with firefighters, we suggest that firefighters’ relatively homogenous, family-like, internally protective and externally respected working groups are the main factors for promoting and maintaining their health and well-being.

Health and well-being have been the subject of different philosophical reflections throughout history. In 1986, the World Health Organization (WHO) defined health as ‘a resource for everyday life, not the objective of living. Health is a positive concept emphasizing social and personal resources, as well as physical capacities. (p, 1). Our understanding of the health and well-being concept is similar to WHOs, since it is formulated with a holistic approach, where health is viewed as a “whole”, including physical, mental and social factors in interaction. In this perspective, health is seen as an ongoing process, constantly created and maintained in a context (Medin & Alexanderson, 2001). In line with this definition, health is to a high degree related to the individual’s context and affected by social relations and work conditions, as well as cultural norms and values (Glozah, 2015; Mirowsky & Ross, 2003). Sarvimäki (2006) has presented a similar Heidegger-inspired description of well-being in which we as human beings are understood as being affected or caught up in our everyday life cycles, including all our doings and non-doings, work, projects and also, anxieties. Eriksson’s (1996) theory of health includes three dimensions: healthiness, freshness and well-being. Healthiness and freshness are described as objective dimensions, and well-being is described as a subjective dimension. This three-fold understanding also harmonizes with WHO’s (1986) definition of well-being, described as an individual subjective experience of health. Wilkinson and Marmot (2003) add to the discussion of defining health as something created and made possible in a social and cultural context. During the last decades health equity, as well as inequity, has been highlighted too (Annandale, 2009; Annandale & Hunt, 2001; Doyal, 2000). WHO has also pointed out the importance of promoting societal change with a view to eliminating gender as a barrier to good health (WHO, 2013).

Firefighting, traditionally a male-dominated occupation—numerically and globally (Baigent, 2001, 2016; Ericson, 2004; Ericson & Mellström, 2016)—is undergoing a change in many respects. This is very much the case in Sweden, a nation that in many contexts are proud of its gender-equality policies, and where, for example, the Swedish Civil Contingencies Agency (Myndigheten för samhällsskydd och beredskap in Swedish, MSB) has worked for many years to improve the gender balance among the group of firefighters (Ericson & Mellström, 2016). However, as Jansson and Grip (2012) argue when analysing the Swedish rescue service organization, there are contradictory conceptions of gender equality and diversity at work at the same time here. Efforts are made that both promote and hinder increased equality. Resistance to interventions and policy initiatives among operating firefighters in Sweden and other countries, especially regarding gender equality and diversity, have been identified and documented by a number of different scholars (Baigent, 2001; Ericson, 2014; Ericson & Mellström, 2016; Hulett et al., 2008). Similar patterns of resistance have also been revealed in the past to proposed changes regarding the organization, or work content, of the rescue services. The same types of struggles have been identified when it comes to the education and training of firefighters (Ericson, 2014; Häyrén Weinestål et al., 2014). Ericson (2011) raises the question about whether the resistance towards change when it comes to gender equality should be understood as a way to oppose the strong external pressures from the Swedish Government, MSB, researchers and, at a local level; politicians and superiors/managers within the rescue services. We find the question valid and would like to argue that the issue of class, among other structuring social dimensions such as age, sexuality and ethnicity intersect, and must also be remembered when analysing the firefighter discourse (cf. Carastathis, 2014). Häyrén Weinestål et al. (2014) base their explanation of the resistance to change within the firefighter discourse primarily on the masculinity constructions linked to the occupation. They further describe an emphasis on bodily competencies in firefighting and strong “bromance” relationships between male members of the group, where a certain, “right” kind of masculinity in the context of rescue services has been developed throughout the years. According to Baigent (2001, 2016), one explanatory key to why women are excluded from the rescue services is the conflation of heroism, masculinity and firefighting. Like Jansson (2016), we claim that there is still a need to explore and examine the gender power structures within the firefighter discourse, and in particular the reasons why changes happen at such a slow pace, even if we know a great deal about what inequality patterns look like, and the need to change them (Ericson & Mellström, 2016).

The need to understand firefighter’s resistance to change within the firefighter discourse better is also a result of our previous analyses of health-promoting factors among firefighters that were conducted during earlier stages of this research project (Jacobsson, Backteman-Erlanson, Brulin & Egan Sjolander, 2020, submitted manuscript). In that work, we identified dominant or recurrent health-promoting themes in the firefighter discourse, such as a strong sense of the firefighter community, opportunities for physical exercise during work, the balance between emergency and station work, clarity of professional roles, peer support and tolerance, as well as the role of helper or “the hero”. We also got a good sense of the so-called “other side of the coin”, namely factors that were framed by firefighters as *hindrances* to their health and well-being at work. The rationale for this article is to further explore the meaning and importance of these different forms of resistance among the workforce in the firefighter discourse. The identified recurrent themes could be grouped into three. One concerns *diversity*, especially regarding gender and ethnicity within the rescue services; another is *preventive work*, or more precisely the relation between preventive and emergency work; and the last concerns the training or *education* of firefighters. Apart from being identified as articulations of resistance, these dominant themes have in common that they deal with proposed or recent changes of the current occupation and work culture of firefighters, formulated by politicians and articulated in different kinds of policy initiatives and legislation. Further, the resistance expressed by the firefighters is, therefore, to a large extent, a reaction to external pressures, e.g. from politicians or national authorities, on the rescue services.

The occupational setting and the organization are pivotal for understanding health-related aspects of firefighting. The working conditions differ significantly between full-time and part-time firefighters, since the latter have another occupation as their main workplace. In this study, we only focus on full-time firefighters. The work that firefighters undertake is generally characterized as high-stress, high-risk and low-control. The emergency job skills and work performance required by firefighters in order to handle both emergency care tasks, and fire suppression, are very demanding (Landen & Wang, 2010). Emergency work is characterized by time urgency, needs accurate decision-making, involves threats of injury and/or death to self and others, includes witnessing deaths and injuries, and requires that one transfers news about tragedy to close relatives and friends of victims (Del Ben et al., 2006; Jacobsson et al., 2014). However, even if pivotal and often in focus, it is important to keep in mind that this operational, highly demanding emergency part of the work only constitutes a smaller part of a firefighter’s total working time. MSB (2009a) estimates that on average, emergency work accounts for approximately 5% of a firefighter’s time at work. The other 95% of the work, so-called contingency time, consists of different types of preparatory activities, such as daily physical exercise, equipment and vehicle reviews, plus repeated group exercises to tackle potential hazardous events. Different forms of preventive work, like school visits and open days at the rescue station for the general public etcetera, are also part of contingency time. There is, on the whole, a relatively good opportunity for firefighters to recover from emergency work during the contingency time. The conditions vary of course from time to time, and for different individuals, and depend on the incidence frequency at any particular rescue service station.

In Sweden, the local rescue services are organized and run by the municipalities. In total, they employ more than 5,000 full-time firefighters and almost 11,000 part-time (MSB, 2015a). MSB is the national agency responsible for civil protection, public safety and emergency management, such as prevention, preparedness and response in case of emergencies (MSB, 2009b). One of MSB’s tasks is also to support the local rescue services in the country. For example, the agency offers training and education in different areas, including training of firefighters for the municipal rescue services. The Swedish Government steers MSB and the local municipalities via a law and a body of instructions (SFS, 2003:778). The instructions specify MSB’s responsibilities and tasks (MSB, 2009b).

## Aim and research questions

The aim of this study is to analyse how recurrent health hindrance themes in the firefighter discourse, identified by firefighters themselves, relate to a set of policies about diversity, preventive work and education of firefighters. The intention is further to discuss the implications of these policy initiatives and the resistance against them in terms of firefighters’ health and well-being at work.

The first research question deals with the dominant health hindrance themes of resistance to change that have been identified as recurrent and common within the firefighter discourse. The second research question concerns the identification of a strategic sample of relevant policy initiatives or directives that deal with these changes of firefighters’ work, and the third research question is about understanding how they relate to each other since it is important to better understand what constitutes these ongoing discursive struggles for, or against, change. The fourth and final research question is about understanding the wider impact of the studied firefighters’ discourse, and in particular the health and well-being of firefighters.

Using a critical discourse analytical (CDA) framework when studying firefighters’ health, enables us to understand how firefighters themselves, both individually and collectively, make sense of their health and well-being (Aho et al., 2019; De Jong et al., 2019; Fairclough, 1989, 1995a; Lövenmark et al., 2018). It also creates an opportunity to learn more about what factors in their work culture that they identify as hindrances to their health. CDA provides both a methodology and a theoretical framework for integrating different analytical levels, like the individual account and the societal context (Egan Sjölander & Gunnarsson Payne, 2011). Furthermore, with CDA we can examine how social forces and struggles may both enable and limit the opportunities to adopt well-being and health among firefighters.

## Method

In this study, as is common in CDA research, we have made use of a combination of methods in both the data collection and analysis (Meyer & Wodak, 2001). Firstly, the study is based on FGDs and individual interviews with firefighters (Jacobsson, Backteman-Erlanson, Brulin & Egan Sjolander, 2020, submitted manuscript). These have been transcribed verbatim (resulting in 128 pages of spoken text), and analysed using text analytical tools to identify common themes in firefighters’ articulations about their health and well-being (Egan Sjölander, 2004). Secondly, a strategic sample of policy documents, collected from a variety of contexts, specifically aiming for change and relating to these articulated themes of resistance, have been analysed with a similar textual analytical approach (cf. Hakimnia et al., 2014).

### Participants and procedure

Firefighters from three different stations, spread across the country and varied in size, were invited to take part in either a FGD (25 participants), or an individual interview (3 participants) about health and well-being at work. The inclusion criteria used were that the participant was being responsible in emergency positions in operative duty as a full-time employee with at least 1 year’s experience. For the individual interviews, a convenience sample was performed. All firefighters in the work shift team that were on duty the actual FGD day from the studied rescue service stations were invited by us directly to participate and informed both orally and by letter about the study. As far as we know all of them available at work or “on duty” the actual day of the FGD also did take part voluntarily. A total of 28 firefighters (four women and 24 men) aged between 28 and 63-years-old participated in FGDs and individual interviews. On average they had worked 21 years in the field. The first focus group had 10 participants, the second six, and the third nine. Two of the participants in the individual interviews were currently working as firefighters while the third one had changed career path even if still employed by the municipality. One of the female firefighters said in an individual interview that she had changed career 1 year ago, since “ … *I could not keep up all that … or match all of these expectations”*. We applied the “information power” concept as defined by Malterud et al. (2016) in their guidance for qualitative research regarding sufficient size of a sample. This meant that we collected empirical material until “information power” was achieved, meaning that recurring themes in the dialogues with firefighters appeared and this despite them being held at different rescue stations in terms of size and geographical location. All three focus groups took place at the respective rescue station, and two of the individual interviews by telephone. The last individual interview was conducted at the participant’s current workplace. All the FGDs and one individual interview were conducted by two researchers, one moderator (AJ) and one observer/secretary (SBE), and two of the individual interviews were done by one interviewer (AJ). All FGDs and interviews were audio-recorded and each lasted between 75 and 97 minutes. The purpose of the study was introduced initially, together with the issue of informed consent. All participants approved the FGD and interview arrangements and gave their consent before taking part. A semi-structured interview guide was used in all cases, containing a list of topics and open questions regarding health and work-related issues (cf. Morgan, 1996) To enable broad participation in the FGDs, a key function of the researcher acting as chair was to make sure that each participant had a chance to speak and share their views on the discussed matter regardless of, e.g., age, number of years in the profession or role/responsibility within in the group. The accompanying role of the observer/secretary during the interviews was also to facilitate this process of broad involvement in the dialogues and to, e.g., notice if someone had tried to raise their voice previously, but not yet spoken.

### Policy documents

**Figure 1.**
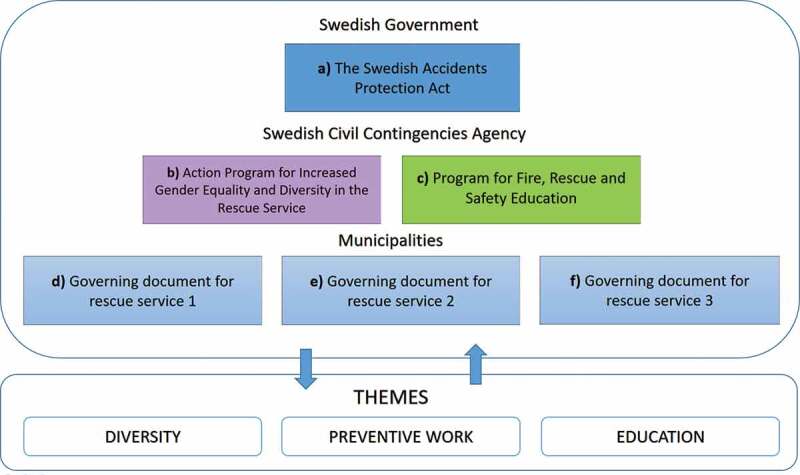
Model of policy initiatives and legislation that relates to hindrance themes in the firefighter discourse.

After retrieving what hindrances firefighters framed as threats to their health and well-being at work, the next step was to identify which policy initiatives promoting change that were relevant to and corresponded to these expressions of resistance among the workforce. More specifically, the hindrance themes are diversity, preventive work, and education. This process of placing (FGD and interview) texts into a wider (policy) context, has been a comprehensive analytical exercise in itself, starting in the present and delving gradually into the past (Egan Sjölander & Gunnarsson Payne, 2011; Fairclough, 1995a). It included scanning and reading a variety of official documents produced at all levels within the Swedish rescue services, such as strategy plans, action programmes, development reports, and steering documents. MSB’s reports and current legislation in the area have also been of importance. The policy documents that finally were selected for closer analysis represent a strategic sample of written materials that can be linked to the themes highlighted in the FGDs with firefighters, and that we evaluate as important within the organization and firefighter discourse (cf. Erlandsson et al., 2016). The documents, both policy initiatives and legislation, were identified at various places and levels in the rescue service organization, and ranged all the way from state level, the Swedish Government, MSB, and further down to local municipalities that the studied rescue services are part of (see Figure 1). The following strategic sample of policy documents were chosen and analysed in more detail: (a) *the Swedish Accidents Protection Act* (SFS, 2003:778); b) *Action Programme for Increased Gender Equality and Diversity in the Rescue Service* (MSB, 2009c); c) *Program for Fire, Rescue and Safety Education* (MSB, 2016); d) *Governing Document for Rescue Service I;* e) *Governing Document for Rescue Service II*; and f) *Governing Document for Rescue Service III* (Figure 1). Further demographic information in the form of references to the local governing document (d), (e) & (f) for each rescue service is not given due to identity protection of these participants and groups. These policies form part of the wider context or surrounding socio-cultural practice that the discursive practice at each rescue service station is influenced by, hence the importance of studying them (Fairclough, 1995a). All analysed documents (policies and directives) are different kinds of public acts and therefore also, in accordance with Swedish legislation, easily available for us as researchers.

Firstly, regarding gender equality and diversity as health hindrances, we started our search at MSB’s website since the authority has worked for a long time with these issues in relation to firefighters’ work environment, and consequently has initiated a series of policy initiatives and projects dealing with gender equality and diversity in the nation’s rescue services. When MSB presents its work regarding gender equality and diversity the authority states that

The MSB is pushing forward and supporting municipalities in their efforts to increase the number of women in local fire brigades, as well as the number of individuals of a non-Nordic ethnicity. (MSB, 2015b)

The same MSB website page is also the first hit that emerges when we searched for documents that deal with diversity. This website presents an overview of the diversity work directed towards the rescue stations in Sweden, and additionally guides us further to a five year; *Action Program for Increased Gender Equality and Diversity in the Fire Service* (MSB, 2009c). The action program was included in our sample since it is described as the most comprehensive action program about gender equality and diversity initiated by MSB. This policy document explicitly tries to push forward equality, and change the traditional, male-associated work culture of firefighters. Gender equality is generally seen as an important aspect of working life in Sweden, a country that is often ranked as one of the world’s most gender equal countries in the world (World Economic Forum [WEF], 2016).

When it comes to the second theme of health hindrances, preventive work, the local governing documents for each of the studied rescue services have been chosen for closer analysis, since it is the municipalities that are responsible for and organize the local rescue services organization. The *Governing document for rescue service I* (d), *II* (e) and *III* (f) were found at the respective municipality website for each rescue service station. These local steering documents are in turn linked to legislation or, a) the *Swedish Accidents Protection Act*, (SFS, 2003:778). This legislation put emphasis on preventive work within the rescue services organization that traditionally and historically has been focused on emergency work. This legislation can be related to the resistance themes regarding change that are formulated within the firefighter discourse, and in particular the reluctance to carry out preventive work.

Thirdly, regarding the education theme, the selected policy document that we have included in the sample for closer analysis is the steering document called; “*Program for Fire, Rescue and Safety Education”* (MSB, 2016), which includes intentions, descriptions of content, and expectations on the revised educational program for firefighters that MSB has run since 2003. The same program constitutes the main object for critique from firefighters.

Lastly, but not least, the mentioned principal Swedish legislation that governs all work within the nation’s rescue service has been included in the analysed sample because it is evident when analysing the Swedish firefighter discourse that, a) the *Swedish Accidents Protection Act* (SFS, 2003:778) influences both the balance between preventive and emergency work at the rescue service stations, and what the renewed education of firefighters should look like.

## Critical discourse analysis and critical policy analysis

As mentioned, we use CDA inspired by Norman Fairclough (1989, 1995a) to analyse both the texts from the FGDs and the individual interviews with the firefighters, and the strategic sample of policy texts relating to the themes of resistance. Within CDA, speaking or articulation is viewed as a way of acting in the world, as participating in social practices, and a means for making sense. Further, Fairclough (2003) points out that our understanding of the world both forms, and is shaped by, discourse. Fairclough (1995a) has also developed a three-dimensional framework for studying discourses that are widely used by scholars in different fields, the aim of which is to relate three separate levels or forms of analysis to each other. The first concerns the concrete analysis of language or texts (spoken or written) on a micro-level. The second level is about analysing the discursive practice; in other words, processes of text production, distribution and consumption, on a meso-level. The third element analyses discursive events as instances of socio-cultural practice on a macro-level. Applying this three-dimensional framework allows us to analyse each articulation about health and work expressed by the firefighters, as text. These are in turn articulated within a specific context, namely the studied rescue service stations that we understand as the *discursive practice*. The three main topics or themes that are articulated by firefighters as hindrances to their health and well-being in the workplace all relate to different types of change that the organization is undergoing or have been facing recently. One is change regarding diversity in the workforce. Another concerns the balance between preventive and emergency work in the organization, and the third relates to the “new” education of firefighters. In order to understand these changes in a broader context, we have linked the texts to policy documents and legislation that encompass all the above-mentioned themes. We thus include the wider *socio-cultural practice* or “macro” context that affects the firefighter discourse.

The analysis of the policy documents and legislation were not only based on Fairclough’s discourse analytical method, but also inspired by Bacchi’s (2016) critical form of policy analysis. Applying Bacchi’s approach helped us also to identify relevant policy documents and to choose which ones to include in the analysis. We searched for policy initiatives that addressed health hindrances or expressed with Bacchi’s words “the problem” of diversity, preventive work and education. With her “policy problematisation approach”, Bacchi points out the need to critically explore the types of knowledge that shape current governing practices and new policies. And further, it is crucial for the researcher to ask what sort of problem the suggested solution is meant to solve. Policy proposals tend to indicate what and whom is in need of change and this also needs to be critically reviewed. Following Bacchi’s recommendation, we have explored how “*problems*” have been constituted in the specific policy documents about firefighters and firefighting that we have analysed. According to Bacchi, it is important not to view “problems” as given; on the contrary, one needs to think about what is considered to be solved? And further, what consequences does the suggested solution have for the subject positions created and transformed in a particular discourse? In this study, one can relate the resistance to changes expressed by firefighters as reactions to the policy initiatives and legislation at work within the firefighter discourse. The “problems” addressed concern the workforce and work environment of firefighters, which the majority of them, in turn, do not define as such. Linking policy documents and FGDs and individual interviews together enabled us to better understand ongoing struggles in the firefighter discourse, including firefighters’ resistance to the policies’ proposed changes.

## Results

The following section presents the three dominant themes of resistance that the firefighters articulate in relation to changes promoted in policy texts, which the Swedish rescue services are facing and undergoing. As one of the interviewed firefighters describes their work situation; “*We just have to realize that it will not continue to be this good. When I look back on my time as a firefighter these last few inspired years, the employment situation has deteriorated”.*

### Diversity as a hindrance to health and well-being

The most common hindrance to firefighters’ health and well-being at work that is brought up in the FGDs is the pressure to progress towards increased gender equality. During all FGDs, the subject of diversity and in particular gender equality is a recurring topic for discussion. The firefighters express that they experience a strong pressure to change, in combination with a lack of understanding in the organization for their situation.

When it comes to diversity and equality, I have never understood the problem … I know we started this three years ago … we started with this diversity, equality work … but I have never seen the problem. For me, there has never been a problem before. (Focus group III)

A similar pattern is reflected in many of the policies as well. The political process and the work with equality and diversity in the Swedish rescue service have a relatively long history. As early as 1997 the Swedish Rescue Services Agency (what is now called MSB) e.g. gave instructions to local authorities to investigate what measures could be taken to improve the recruitment of female firefighters to the rescue service (MSB, 2009c). This “national” support to local bodies has existed ever since, ranging from various activities and initiatives, to studies, for example, about the firefighter profession and local organizational structures.

A national *Action Program for Increased Gender Equality and Diversity in Municipal Safety* (MSB, 2009c) was launched by MSB in 2009 to promote an egalitarian, diverse rescue services organization. The goal to strive for, according to this program, is “*An equal rescue service that reflects society in general, and this is a prerequisite primarily from a democratic perspective”* [our translation]. The problem the policy intends to solve is “*The culture that maintains the very homogeneous profession, the rescue service, itself creates problems”* (MSB, 2009c, p. 7). The problem is further defined in terms of how many, or rather how few, women in relation to men that are employed as firefighters. In 2009 about 60 women worked as full-time firefighters (1%) in the whole of the country, and 338 women worked part-time (3%). The numbers are described as very low even in comparison with other countries’ fire brigades, and other traditional male-dominated industries. The conclusion that is drawn in the report states “*that women are largely excluded from work on protection against accidents in the civil service”* (MSB, 2009c, p. 7).

The same action program also examined the physical work environment of firefighters, since it is identified as a problem area that has to be solved in order to attract more women. *“Examples of activities in this area may be the acquisition of equipment specifically adapted for women”* (MSB, 2009c, p. 18). Based on our FGDs with the firefighters, they also noted this problem, but from another perspective, as one of them expressed it:

They want to bring in new technology and new equipment that is lighter and more ergonomic, they want to develop and develop, and it is all in a hurry … But it is impossible to develop away that basic fact that firefighting is a physically demanding profession when it gets tough. And that you cannot avoid, of course not. (Focus group III)

Another of the informants confirms this view and says that “*They are building an air castle”* (Focus group III). Even though the action program has diversity in its title, along with gender equality, relatively little attention is directed towards other grounds for discrimination than gender. In the report, diversity is defined as more than a gender issue. Ethnicity, sexual orientation, religion (and other beliefs), disability and age are also mentioned.

However, the work with the action program has focused on gender, ethnicity and sexual orientation. The reason is that it is mainly those who have proven to be problematic to unite with the idea of who can be a firefighter. (MSB, 2009c, p. 7)

Despite these claims, when reading the whole document it is clear that the main problem is understood to be the numeric lack of female firefighters in the rescue service organization. The experience of feeling like an outsider as a woman and not adhering to the firefighter norm, but trying to fit in is articulated in various ways in our interviews, as one of the firefighters explains:

… It also depends on how well you adapt to, how to melt into the group. You must not stand out in any way, be bad in any way. It was quite difficult to do ‘right’ and to be good enough. Because if you performed well then it was no good, and if you were not good at something, it was a disaster. If you did not talk much it was no good, but if you did talk a lot it was no good either. It was very difficult to read the situation and to please everyone. (Individual interview 3)

### Preventive work instead of emergency preparations as a hindrance to health and well-being

The *Accidents Protection Act* (SFS, 2003:778) is the “superior” law governing the whole rescue service organization in Sweden. MSB in turn coordinates all risk and safety work in the country. The agency has also a general responsibility for developing and supporting the nation’s ability to deal with accidents and crises. MSB (2009b) does this in cooperation with municipalities, county councils, government agencies, companies and organizations. At the local level, it is the municipalities that are in charge of organizing the rescue services, usually in collaboration with other principal functions with operative responsibilities. The primary mission of the rescue services as stated in legislation (SFS, 2003:778, 3rd chapter, §1) is to “*ensure that measures are taken to prevent fires and fire-related damage and to work to establish protection against other accidents, without compromising the responsibilities of others”*. All governing documents for rescue services, from the different municipalities that are analysed in this study (d, e, and f) take their “point of departure” in this law (see Figure 1). The heading at the very beginning in one of them states: *“the best accident is one that never happens”* (*Governing Document for Rescue Service III*). The importance of emergency rescue service work is further highlighted, vis-à-vis’ the emphasis on preventive work in all those documents. One thing that differs in between these local policies, however, is how extensively the different parts of the business are described.

When asked, during one of the focus groups (II), about the content and forms of the prevention work that firefighters undertake, the rescue leader responded: *“We have three areas of focus for prevention … ”*. Then he paused a moment before giving more details about them, but at the same time he realized that actually *“I do not remember …, in fact I forgot them all”* (Focus group II). The (only) female firefighter in the group tried to help out:

… our current job description says that we have to produce (read work with prevention, our comment). Prevention is prioritised today. But when production is interrupted by an alarm, then we will work quickly and efficiently, in order to be able to go back into production again. (Focus group II)

Thereafter the group discussion highlighted firefighters’ concerns regarding the organization’s contemporary strong focus on prevention. As one of them explained:

It is worrying, because it will be harder to be prepared for rescue work. I think for various reasons and partly due to the changes in the profession. I can list several things that have got worse. (Focus group II)

The rescue leader agreed and elaborated*: “Now the education of the public is a huge part of our time/ … /kindergarten weeks for example. It takes a whole week to have them with us* (the children, *our comment*)*”* (Focus group II). The current emphasis on prevention is viewed as a change in the profession or work as a firefighter. This new focus, especially directed towards children and young people, is also described in the *Governing Document for Rescue Service II*; it states that “*the biggest difference from before/ … /is that the rescue service is going to work systematically to make people feel better”*. With a vision of what is described as an inclusive society, the rescue services are intending to ensure that people they meet who are feeling ill get help, and that individuals and groups that feel excluded increase their trust in society and its functions. This work aims to improve all people’s potential to live a good life. Safety, trust, social control and cohesion are signs of an inclusive society, including a decreasing number of accidents, according to this policy document. The overall solution to the “problem” in the *Governing Document for Rescue Service II* is further to prevent accidents. Apart from a focus on children and young people, maintenance of a good emergency service organization and prevention of fires are pointed out as prioritized areas in this rescue service’s work. All FGDs repeatedly described the stronger focus on prevention as an inconvenient element and sometimes even as an obstacle to carrying out the operational parts of the work. The discrepancy between what was emphasized during the training to become a firefighter regarding preventive work and the practical work culture at the rescue stations was strongly apparent during work experience periods according to one individual interviews, “*it then became even clearer, very clear, that there was little interest in preventive work”* (Individual interview 2).

### Education instead of skill training as a hindrance to health and well-being

The main law governing the rescue services in Sweden (SFS, 2003:778) also includes instructions regarding the education of firefighters and stipulates that MSB is responsible for their training. The Accident Prevention Training course (Skydd mot olyckor, SMO, in Swedish) was designed in 2003 with the aim of increasing the impact of risk prevention work in the national rescue services. This meant, among other things, an increased degree of academic or theoretical subjects for firefighters, and a broadened professional concept. The two-year, post-secondary level, educational program is designed for future full-time firefighters. The SMO-education replaced the previous educational system within the rescue service where the rescue service organizations themselves selected participants for further training within their own organization. In the cases where these trainees were included in the permanent workforce, they also had to undertake a 15-week, more formal, education. Today, MSB is also responsible for the education of part-time firefighters, who have to undergo a nine-week course entitled Emergency Response Operation program.

One of the incentives to introduce the SMO education program was the need for a more transparent and equal recruitment process to the rescue service. One central objective in the MSB (2009c) action program towards increased gender equality and diversity is also to increase the number of women applicants to SMO. Thereby one hopes to increase the recruitment pool for the rescue service in Sweden. The SMO education follows the *Program for Fire, Rescue and Safety Education* (MSB, 2016). Here the mission to educate firefighters with a broader perspective than just operational emergency work, is visible:

Essential for the education is a pre-, under, and after-perspective approach to accident management, as well as society’s emergency preparedness, focusing on municipal activities. The education also aims at developing a holistic approach to contribute to actors’ common management of social disturbances. (MSB, 2016, p. 1)

The firefighters participating in our study are generally critical towards the new education that firefighters get today. This is a recurring theme that is brought up in all our FGDs. The firefighters believe that the revised education programme has too strong an emphasis on preventive work, at the cost of training all practical skills needed in firefighting. As one of them formulates it: *“They forget that they must have the practical skills, the know-how when it happens, it is not possible to train and practice then”* (Focus group II). Another firefighter reasons in a similar way:

They ignore completely the need for practical skills in this profession. So if they never held a hammer and screwdriver, maybe they are not suited for this profession. Or maybe you need a very long time to get into it/ … /then maybe they need to have extremely much practice time to become good at these things, to hold a screwdriver, or what to do when…(Focus group III)

Another colleague in the same rescue service expresses a similar worry: *“When it really counts, it is when everything is brought to a head, which is when it reveals if you can handle the job. It is impossible to practice then … . when it really counts”* (Focus group III). According to one of the firefighters that had stopped working as such, it was clear already during the SMO education that for those who had a parent or a dad, or someone else they knew who worked in the emergency service, preventive work had a very low status right from the start. And this even if this broadened assignment for firefighters including preventive work was a crucial part of the education (Individual interview 2).

## Discussion

The work of firefighters, traditionally a male-dominated occupation, is undergoing change. This is the situation in Sweden and in many other countries around the world. In this article, we have analysed the resistance to change that firefighters express when discussing their health and well-being at work. In summary, three recurrent themes of resistance are articulated in the studied firefighter discourse. The first one concerns the demand for increased diversity in the workforce. However, all the attention in the discourse is directed towards gender equality, and more precisely the number of women working in the rescue service. The second theme is about the increased focus in the organization on preventive work that firefighters nowadays are expected to undertake. What was previously an option, or a decision based on the free will of a firefighter and his/her team, has now turned into an expectation from other actors in society, not least politicians at national and local levels. The third theme that generates resistance concerns the contemporary education of firefighters, which has become more formalized, centralized and academic compared to previous years. Before the new law was introduced in 2003, practical skills in emergency situations constituted the prime focus when training firefighters.

The dominant resistance themes that firefighters formulate when discussing their work and health can further be understood as responses to external pressures and demands from the surrounding society on the rescue services. In this study, we have analysed a strategic sample of policy initiatives that can be related to this reluctance to change within the firefighter discourse. And this in order to get a better understanding of what types of “new” demands are directed towards the firefighter profession and its current organization, and how they are framed. We have also noticed that these different policies are intertwined and linked to each other, like the emphasis on preventive work in both the organization and the education of firefighters. Also, one realizes when studying these texts that the call for change, for example, regarding increased diversity in the workforce, is far from new, which shows that changes in these areas are happening at a relatively slow pace. This is also a pattern that is recognized in other male-dominated fields such as forestry (Johansson et al., 2019, SOU 2004).

Diversity, preventive work and education are all examples of pivotal struggles in the firefighter discourse. They engage stakeholders with conflicting views about what is needed in the rescue service and who is “right” to decide about its future. On the one hand you have the “insiders”, a vast majority of male firefighters working in the rescue services around the country who are representing a collective that defends their autonomy and traditions. They are all motivated to do so since they share a strong compassion to serve and guard the community and to guarantee the safety of people and goods. In this respect, workers and rescue leaders within the services speak with a united voice, more or less “as one”. On the other hand you have the “outsiders”, represented by the government, the national authority (MSB), municipalities, politicians at different levels, as well as other actors within Swedish society, which are proud of their gender equality work. Academic researchers, primarily from the social sciences and often consulted by MSB to conduct studies, are also part of this outsider group that from time to time add to the pressure on the nation’s rescue services to change. The resistance within the firefighter discourse that much of previous research, as well as our study, have observed are often framed as a problem deriving from the dominant cultural and normative understanding of the profession. This in turn strongly links firefighting with masculinity and gives firefighters a high, hero-like status in society. The risk with increased diversity, not least more female firefighters, is understood as a loss of this status since it is built around e.g. physical strength. Many studies have further problematized the implications of this dominance of a certain kind of hegemonic masculinity, that not only hinders change, but that also puts constrains on male and female firefighters working in the services today (Baigent, 2001; Chetkovich, 2004; Ericson, 2011; Ericson & Mellström, 2016; Häyrén Weinestål, 2014; Olofsson, 2012). The wider implications of the studied firefighter discourse can be identified at both a group and an individual level, and as far as our results show, the most negative and devastating effect can be found among firefighters who have been excluded and not accepted within the group. Those people, the individuals that do not conform to the norm, give witness to harsh treatment from their peers. This impression is as strong as the community sense that the vast majority of firefighters’ experience and enact on a daily basis. If you belong, if you are part of the collective, then you are also cared for by the other firefighters.

In addition to these explanations relating to gender, it is also fruitful in our view to understand articulations of resistance in terms of health and well-being among firefighters. We claim that applying such a perspective brings a new understanding to the reasons why change is happening at such a slow pace within the firefighter discourse, or in some cases, not at all. One could simply say, looking at our results, that what is at stake is the health and well-being of the majority of firefighters. This is a group of employees that are relatively healthy, despite high demands and risks associated with their job (Jacobsson et al., 2014). Their well-being, at least partly, derives from a strong sense of belonging to a homosocial group or team where peers take care of each other. This community-sense is further built on similarity, rather than diversity. This is also part of the explanation why it is often tough for females, non-whites, HBTQ-persons and other who are “not similar” persons, to fit in and why it can be so painful to be excluded. The resistance to change within the firefighter discourse is linked both to the hindrances that firefighters identified to their health and well-being, and to the policy initiatives that tries to open up and influence the future of firefighting. The most important challenge to tackle in the future, therefore, appears to be how to transform firefighting to be more inclusive and yet maintain the good health and well-being that exists among the vast majority of firefighters. This can never be done successfully in an organization if the employees resist such changes. They need to support or promote such policies. One implication from this research is that the identified resistance to change, and its association with the health and well-being of firefighters, needs to be looked at and discussed more at depth within the Swedish rescue service in order to be surpassed. And this should be done not only by policymakers and management at national and local level, but preferably include all employees. Other lessons learned from this analysis, with implications also regarding the future education of firefighters, are that diversity needs to be understood in both theory and practice as something much more than gender distribution or simply counting numbers of male and female firefighters within the workforce. The dominant polarized view in firefighter discourse which places emergency work contra preventive work is also problematic since they are interdependent. The increased external pressure on the rescue service when it comes to preventive work is further part of a more general trend in society of improved productivity in our workplaces. The same can be said about the training of firefighters and the increased demand for academic knowledge contra practical skills. These issues are not only pivotal matters of health and well-being in firefighter discourse, they are also important subjects for future research.
